# Evaluation of Selected Essential Elements in Khalas Dates from Date Palm Determined by Inductively Coupled Plasma-Mass Spectrometry

**DOI:** 10.1155/2019/7619692

**Published:** 2019-06-02

**Authors:** Eid I. Brima

**Affiliations:** ^1^Department of Chemistry, College of Science, King Khalid University, Abha 61413, Saudi Arabia; ^2^School of Allied Health Science, Faculty of Health and Life Sciences, De Montfort University, The Gateway, Leicester LE1 9BH, UK

## Abstract

In terms of nutrition, dates are an important commodity because they are a source of carbohydrates and minerals. Saudi Arabia is the second largest producer of dates worldwide. Khalas is the tenth most popular date type in the Kingdom of Saudi Arabia (KSA), but only limited information related to the levels of essential nutrients in Khalas dates is available. The concentrations of Mn, Cu, Zn, and Se were determined using inductively coupled plasma-mass spectrometry (ICP-MS). The average concentrations in wet weight were as follows (mg/kg): Mn (2.90 ± 0.54), Cu (1.78 ± 0.64), Zn (1.72 ± 0.42), and Se (0.10 ± 0.06). The calculated intakes (*μ*g/kg bw day) per 100 g dates for each element were as follows: Mn (4.14), Cu (2.54), Zn (2.46), and Se (0.14), which represent 0.14%, 0.51%, 0.25%, and 0.2%, respectively, of the provisional maximum tolerable daily intake (PMTDI) recommended by the EFSA/WHO. It was found that levels of the analysed essential elements in up to 100 g of Khalas dates do not exceed the level set by the EFSA/WHO.

## 1. Introduction

Dates (*Phoenix dactylifera L*.) have nutritional importance and have a fundamental role in the economy of the Kingdom of Saudi Arabia (KSA). Therefore, in-depth analysis of dates is necessary to enhance scientific knowledge related to this important food commodity. This study focuses on a popular type of date in KSA, which is called Khalas. Khalas has a sticky texture with a soft and sweet taste and is usually consumed with Arabic coffee. Khalas is mainly cultivated in Qaseem and Al Kharj. Khalas dates are produced in large quantities in the eastern provinces of the KSA, especially in Al Ahsa. Khalas dates have a sweet taste and are popular among consumers in the KSA [1].The average weight of a nonpitted date is 3.63 g, with 137 dates having a weight of approximately 500 g. Therefore, Khalas dates are rather small and can be classified as cane sugar variety because their moisture content (22.5%) is <26% [2].

Essential elements are important for human health [3]. Therefore, the mineral content of dates must be compatible with standard guideline values.

In this study, we measured the zinc (Zn), manganese (Mn), copper (Cu), and selenium (Se) contents of Khalas dates. Zn and Cu are essential elements involved in various biochemical functions in humans, which are necessary for good health. In particular, Zn is required for the function of more than 200 enzymes, some of which are important for protein and nucleic acid syntheses [4, 5].

Mn is necessary for bone health in humans and plays important roles in blood sugar regulation, carbohydrate and fat metabolism, and calcium absorption. Mn is also classified as an antioxidant that fights free radicals and is involved in proper nerve and brain functions [6]. Se acts as an antioxidant along with Vitamin E to remove free radicals. Both the immune system and thyroid function must be intact for Se to function appropriately [7].

Different studies were performed to measure the mineral contents in dates. A recent study conducted nutritional analysis of dates from Bangladesh and showed that the concentration ranges of Mn, Zn, Cu, and Se (%mg) were 0.85–1.1, 0.69–0.72, 0.32–0.36, and 0.22–0.31, respectively [8]. A previous study that considered five date cultivars (Ajwa, Beid, Burni, Rabia, and Safawi) showed that potassium was the predominant macroelement (0.185-1.51%) in all investigated varieties of dates. Zn and Cu were reported to be in the ranges 27.5–72.70 and 4.95–6.25 ppm, respectively [9]. A previous study on date fruits from Iraq showed concentration ranges for Mn, Cu, and Zn (mg/kg) of 51.4–58.6, 25.4–28.9, and 7.4–18.2, respectively [10].

This study was performed to assess the essential element content in Khalas dates consumed in Asir region (Abha and Khamis Mushayt cities) imported from different locations in the KSA using inductively coupled plasma-mass spectrometry (ICP-MS). An additional aim was to compare levels of essential elements in Khalas obtained from different locations. Moreover, our final aim was to calculate the dietary intake of each element after consumption of several date fruits based on a single date fruit intake.

## 2. Materials and Methods

### 2.1. Khalas Dates Collection and Preparation

In total, 10 samples (duplicate) of five types of dates were purchased from retailers (local markets) in Asir province from Abha and Khamis Mushayt cities. Khalas dates originating from different locations in the KSA, including Najran, Wadi Al Dawasir, Al Kharj, Al Qaseem, and Al Ahsa, were obtained. The average weights of a single date fruit without pits were as follows: 6.11 ± 1.18, 6.64 ± 0.94, 5.49 ± 1.47, 6.21 ± 1.65, and 5.49 ± 1.11 g from Najran, Wadi Al Dawasir, Al Kharj, Al Qaseem, and Al Ahsa, respectively. Therefore, the average weight of a single Khalas date from five different locations is 5.99g.

All collected samples were washed with deionised water and dried at 25°C before the pits were removed. The sliced dates from each sample were weighed and dried in an oven at 80°C overnight. The average moisture percentage (%) in all samples was 22.53% with a range of 19.22–31.18%. The dried samples were pulverised using a coffee grinder. The powdered samples were stored in small zip bags in a dry place for the subsequent digestion process and measurement by the ICP-MS. Digestion, measurement, and chemical reagents were the same as previously described [11]. For digestion purpose 0.5 g of a powdered dried date was mixed with 4 mL of conc. HNO_3_ and 2 mL of H_2_O_2_, and the mixture was digested for 40 min using a Multiwave 3000 microwave sample preparation system. The digested sample was made up to 10 ml with deionised water

### 2.2. Quality Control

The limits of detection (LODs) and quantification (LOQs) were calculated by 10 measurements with 1% HNO_3_. The calculations were as follows: LOD = 3 × SD and LOQ = 10 × SD. A continuing calibration verification (CCV) was used as a quality control (QC) test for each run. This was performed by testing 20.0 *μ*g/L of a mixed standard containing all measured elements after each set of five runs. A spiked sample with a specific concentration (20 *μ*g/L) of each measured element in a mixture was analysed.

### 2.3. Quality Assurance

For quality assurance a certified reference material (CRM), Rice Flour-Unpolished, Low Level (Cd) (No. 10a NIES), was measured.

### 2.4. Statistical Analysis

Statistical analysis was performed for all date (Khalas) samples using one-way Analysis of Variance (ANOVA) in SPSS (version 22) to compare means. The statistical significance was defined as P < 0.05 with a 95% confidence level.

## 3. Results and Discussion

### 3.1. Quality Control

The LOD values (*μ*g/L) were as follows: Cu (0.35), Se (0.043), Zn (1.76), and Mn (0.11).The LODs in this were lower for other kinds of dates in the literature [12]. The LOQs (*μ*g/L) were as follows: Cu (1.18), Se (0.14), Zn (5.87), and Mn (0.38).

The averages recoveries after the CCV process for each element in a single session after each of the five runs were as follows: Cu (93.95%), Se (102.75%), Zn (81.95%), and Mn (97.55%).

The accuracy of the proposed method was evaluated by determining the recoveries of the analytes by spiking experiments. Recoveries from the spiked sample for each element were as follows: Cu (80.13%), Se (82.69%), Zn (87.57%), and Mn (78.79%).

### 3.2. Quality Assurance

The concentrations (*μ*g/g) of the measured results of the CRM (No. 10a NIES) were as follows: Cu (3.22±0.08), Se (0.06±0.002), Zn (29.47±0.35), and Mn (2477±0.21), and the certified values were 3.5±0.3, 0.06, 25.2± 0.8, and 34.7± 1.8, respectively.

### 3.3. Levels of the Measured Essential Elements in Khalas Date Fruit Samples

**Figure 1:**
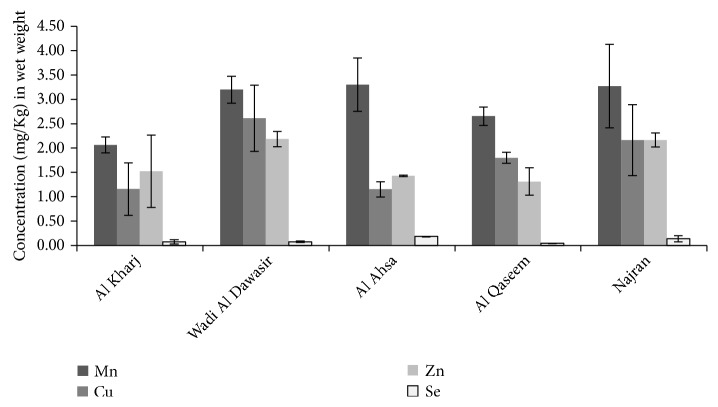
Average concentrations (mg/Kg) of Mn, Cu, Zn, and Se in wet weight Khalas dates from five locations in the KSA. The values are means ± SD. The standard deviations are represented by vertical bars.

Concentrations (*μ*g/g) of the measured elements in wet weight are shown in Table 1 for 10 date samples. The highest concentration was reported for Mn in all samples, whereas the lowest concentration was Se in all samples. The wet weight concentration was calculated from the dry weight concentration as follows: wet weight concentration = (mass of dry weight / mass of wet weight) x dry weight concentration.  Figure 1 illustrates average wet weight concentrations of Mn, Zn, Cu, and Se from the five sources (Najran, Wadi Al Dawasir, Al Qaseem, Al Kharj, and Al Ahsa) of Khalas dates. As shown in Figure 1, the Mn concentration was the highest of all tested elements in all date samples followed by Cu, Zn, and Se in order. The amount (*μ*g) of each element in a single date fruit was calculated by multiplying the wet weight concentrations of ten samples (Table 1) for each element by the average weight of a single date fruit (5.99 g). The calculations were as follow: Mn (5.99g x 2.90 *μ*g/g = 17.37 *μ*g), Cu (5.99g x 1.78 *μ*g/g = 10.66 *μ*g ), Zn (5.99g x 1.73 *μ*g/g = 10.33 *μ*g ), and Se (5.99g x 0.10 *μ*g/g = 0.59 *μ*g ). A single date fruit from each location provided the following elements in decreasing order: Mn > Cu > Zn > Se. We compared the reference values [13–15] of dietary intakes related to microelements, which are as follows: PMTDI of Mn and Cu is 0.5 and 1mg/kg bw per day, respectively, while the PMTDI of Zn is 3mg/day and Se is 70*μ*g/day. We considered a consumption of 100 g of dates, with body weight equal to 70 kg for adult individual. The calculated values (*μ*g/100g) were as follows: 290, 178, 172, and 10 for Mn, Cu, Zn, and Se, respectively, while in terms of *μ*g/kg bw day, the values were as follows: 4.14, 2.54, 2.46, and 0.14 for Mn, Cu, Zn, and Se, respectively. The percentage of each element of the PMTDI, in a decreasing order, is as follows: Cu (0.51%)>Zn (0.25%)>Se (0.2%)>Mn (0.14%).

The average concentrations of Se in a previous study ranged from 1.48 to 2.96 *μ*g/g in the studied samples from the KSA [16]. Herein, we report very low concentrations compared to those of the previous study. However, this study is consistent with the findings of another previous study as we found that dates cultivated in the eastern province (Al Ahsa) contained the highest Se content compared to other samples from different locations. The dates from Al Ahsa showed the highest levels of all elements compared to those of the Khalas dates from the other locations. Previously [17], Khalas dates from the United Arab Emirates (UAE) showed concentrations for Cu (2.8 mg/kg) and Mn (3.0 mg/kg) similar to those reported here. In the previous results, Zn content (14.1 mg/kg) was very high compared to the results of this study, which could be due to the growth soil or packaging process.

A recent study from Bangladesh showed similar trends for concentrations of essential elements in the dates from Al Ahsa and Al Kharj. The trend of that study was Mn > Zn > Cu > Se in three varieties of dates (Trounja, Lagou, and Gounda) from KSA. The ranges of percentages (mg%) were as follows: Mn (0.85–1.1) > Zn (0.69–0.72) > Cu (0.32–0.36) > Se (0.22–0.31) [8]. This indicates that the date fruits show similar overall trends in essential elements content independent of the species and country of origin. Moreover, the various countries, fertilisers, irrigation systems, and soil showed no impact on this trend. Thus, we can conclude that the palm dates absorb the essential elements in a similar manner regardless of the country of origin. In a previous study [18], the nutritional status of the date palm was evaluated. Amino acids, proximate composition, and minerals (including Na, K, Mg, Ca, Fe, P, and Zn) were investigated. Compared to our results, the authors found higher levels of Zn (50.24 ± 1.21 mg/100 g). This high Zn value could be due to the different geographic origin, soil, or irrigation water. In a recent study [19], the elemental concentrations were determined in Segae dates from Al Kharj, KSA. The previous study showed trends that were similar to this study for the essential elements with the order of Mn > Cu > Zn, with average concentrations of 53.65, 1.21, and 0.40 mg/kg, respectively. Compared to our results, the Cu contents reported previously were similar, the levels of Zn were lower, and Mn concentration was considerably higher. A review [20] combined the results of various studies for more than 27 types of dates, including Khalas, to determine the concentrations of Zn and Cu. The ranges of Zn (0.0019–0.013 mg/kg) and Cu (0.0026–0.0097 mg/kg) in terms of dry weight were very low compared to the results reported herein. This could be due to industrial revolution in the last 28 years in the area. Therefore, the industrial growth could contribute to contamination in the whole area, which may have its effect on the higher elemental concentrations reported herein. The review results were interesting, which could have been considered as baseline contamination at that time. Thus, we conclude that contamination has increased by >600 compared to those 28 years ago when that previous study was conducted (136–683 times for Cu and 183–906 times for Zn). A Sudanese study [21] reported the chemical compositions of five cultivars (Red Gau, Barakawi, White Gau, Black Gau, and Gondeila) of palm dates. The decreasing order of elemental concentrations was as follows: Zn (0.36 mg/kg) > Cu (0.31 mg/kg) > Mn (0.30 mg/kg). Zn and Cu followed the same order as shown herein and these results were very similar among the different cultivars. The concentrations of different elements were low compared to our results. This is likely due to different types of investigated date fruits and the country of origin.

## 4. Conclusions

Based on the results presented herein, we can conclude that Khalas dates are a good source of essential elements, including Zn, Cu, Mn, and Se. Differing levels of these essential elements likely reflected the different areas of cultivation. Consumption of seven Khalas dates per day or hundred grams will contribute to levels of these essential elements that will not exceed the guidelines set by WHO. Khalas date fruits provide the measured essential elements in low percentages (0.14–0.51%) of the PMTDI recommended by EFSA/WHO, where Cu was the highest and Mn the lowest.

## Figures and Tables

**Table 1:** Mean concentrations (mg/Kg) of elements (Mn, Cu, Zn, and se) measured in ten samples, two samples from each source: Al Kharj, Wadi Al Dawasir, Al Ahsa, Al Qaseem, and Najran in wet weight samples. The results are presented as means ± SD (n =3).

Sample name	Mn	Cu	Zn	Se
Khalas Al Kharj-1	1.95±0.06	0.78±0.03	1.00±0.02	0.10±0.09

Khalas Al Kharj-2	2.18±0.11	1.54±0.11	2.05±0.15	0.04±0.11

Khalas Wadi Al Dawasir-1	3.39±0.07	3.10±0.09	2.30±0.06	0.09±0.12

Khalas Wadi Al Dawasir-2	3.00±0.08	2.13±0.02	2.08±0.06	0.06±0.14

Khalas Al Ahsa-1	2.92±0.22	1.26±0.01	1.42±0.04	0.19±0.19

Khalas Al Ahsa-2	3.69±021	1.04±0.06	1.44±014	0.18±0.21

Khalas Al Qaseem-1	2.79±0.09	1.88±0.14	1.12±0.07	0.02±0.25

Khalas Al Qaseem-2	2.52±0.14	1.72±0.18	1.51±0.16	0.03±006

Khalas Najran-1	2.66±0.11	1.64±0.08	2.27±012	0.09±028

Khalas Najran-2	3.88±014	2.68±014	2.06±003	0.18±013

*Average*	*2.90*	*1.78*	*1.73*	*0.10*

## Data Availability

The data used to support the findings of this study are available from the author upon request.
